# The presence and absence of gender and intersectionality in the 2023 NDIS review: a content analysis

**DOI:** 10.1186/s12939-025-02441-2

**Published:** 2025-05-19

**Authors:** Diana K. Piantedosi, Raelene Wilding, Maya G. Panisset, Léna I. Molnar, Chloe Bryant, El Gibbs, Anne-Maree Sawyer

**Affiliations:** 1 Women with Disabilities Victoria, Melbourne, Australia; 2 Department of Social Inquiry, School of Humanities and Social Sciences, La Trobe University, Melbourne VIC, Australia; 3 Melbourne Medical School, The University of Melbourne, Melbourne, Australia; 4 School of Health and Social Development, Deakin University, Melbourne, Australia; 5 School of Health and Rehabilitation Sciences, University of Queensland, Brisbane, Australia; 6 Disability Advocacy Network Australia, Marrickville, Australia; 7 School of Allied Health, Australian Catholic University, Ballarat, Australia

**Keywords:** Gender, Chronic illness, NDIS, Intersectionality, Lived experience

## Abstract

While a world-leading initiative, Australia’s National Disability Insurance Scheme (NDIS) has received criticism for its design and implementation. In particular, gender inequality in access to supports and services remains a significant issue. Was the 2023 NDIS Review successful in addressing the persistent problem of gender inequality? To answer this question, this paper presents a content analysis of key documents produced through the 2023 NDIS Review, to investigate whether and how gender inequality was acknowledged and integrated into the Review’s recommendations. The analysis assessed the frequency and conceptualisation of gender-related terms and of the Review’s preferred term, ‘intersectionality.’ The analysis found that the Review documents have limited references to gender-specific terms, often replacing them with ‘intersectionality’. However, this preferred term lacked an explicit definition and was operationalised inconsistently. Implied meanings were often diluted from the conceptual origins of intersectionality. This means that gender inequalities have been largely ignored in both the findings and recommendations. We conclude that the gendered foundation of issues is obscured by diluting interpretations of ‘intersectionality’ to the level of individuals or groups, which sideline systemic critique. Importantly, our article highlights the need for policy makers and researchers to operationalise the term ‘intersectionality’ deliberately and consistently.

## Introduction

In 2013, the Australian Government implemented the National Disability Insurance Scheme (NDIS) as the key legislative mechanism to meet its obligations to the United Nations Convention on the Rights of Persons with Disabilities [39]. The NDIS was designed following extensive consultation with the Australian disability sector. The aim was to provide a system that fosters choice and control and broadens the opportunities and supports for people with disabilities^1^ [34]. However, a growing body of evidence, including a recent NDIS Review [28], indicates persistent gender inequalities in access to NDIS supports.

Although disability prevalence in Australia is similar for males (21.0%) and females (21.8%) [1], there is a significant gender divide in disability support: 62% of NDIS participants are male, and only 37% are female [26]. The gender bias in autism diagnoses [16, 31, p.19] provides a partial explanation of this gap [44]. However, research indicates that other factors, notably gender norms and systemic gender biases, are also significant. In the context of significant gendered challenges, the recent NDIS Review [29, 30] (hereafter referred to as ‘*The Review’*) was seen by some in the disability sector as a welcome opportunity to address evidence of a gender divide in disability supports [42]. Advocacy groups received assurances that it would include a gender equality strategy, perceived as a necessary step forward in ensuring that disability policy and practice would reduce patterns of gender disparity and inequality [46]. In that context, this paper addresses an important question: has *The Review* fulfilled its promise to address the persistent problem of gender inequality?

The paper is organised into four parts. Sect. "Background" outlines the background of the NDIS, and Sect. "Women are underserviced by the NDIS" discusses the limited scholarship addressing women being underserviced by this system. Sect. "The [2023] Review" summarises the history of reviews of the NDIS culminating in the present review. We note that *The Review* prioritised intersectionality rather than gender as the foundation of its analysis of marginalisation in disability support access. In the absence of a definition of intersectionality in either the text or the Glossary of *The Review*, "Intersectionality" outlines the concept of intersectionality and its connection to gender inequality. This extended theoretical foundation is necessary to establish the directive component of our approach to content analysis.

Sect. "METHODS" describes the method: a content analysis of key documents produced through *The Review*. We investigated the frequency and meaning of ‘intersectionality’, as well as other gendered terms. Sect. "FINDINGS" then outlines the findings, offering a detailed analysis of the ways in which both intersectionality and gender are considered throughout *The Review*. The "DISCUSSION" Sect. concludes that gender inequalities have been largely ignored in the findings and recommendations of *The Review*. This has significant implications for Australia’s disability sector, which remains in urgent need of a gender equality strategy to ensure that any reform of NDIS policy meets the needs of all people with disabilities. Importantly, our findings have far-reaching implications for policy makers and researchers globally, highlighting the ways in which inconsistent or misunderstood conceptualisations of intersectionality can obscure the systemic origins of issues, create new forms of disadvantage, and contribute to further marginalisation.

### Background

While the NDIS is rightly seen as a progressive, world-leading legislative framework, it has received ongoing public criticism since its inception. After the initial piloting of the NDIS in 2013- 2015, the Scheme underwent numerous reviews. The 2015 review of the NDIS Act made 33 recommendations, noting the need for greater clarity in how disability requirements are intended to operate for people with chronic illness and improved administration practices [17]. It also recommended that the language used should more accurately reflect the lived experiences of people with disabilities, using principles of co-design [17].

Another review was undertaken in 2019 to identify further opportunities to improve the Scheme and remove legislative barriers for participants [38]. Again, this review acknowledged unresolved tension over the role of the NDIS in supporting disability related to chronic health conditions and recommended ‘detailed policy work’ on this issue as a ‘priority of governments’ [38, p.36]. Likewise, this review concluded that the NDIS was difficult to access, and that many participants found it hard to understand the processes. It also indicated that staff of the scheme administrator, the National Disability Insurance Agency (NDIA) had a poor understanding of the lived experiences of people with disabilities [38].

The repeated message is one of rising concern regarding the Scheme, inconsistencies in decision making around approved supports, and an urgent need for improvement. Some noted a conflict between the government-administered and market-driven approaches embedded in the NDIS [40]. ‘Empowering’ people with disabilities under this marketised system relies on the assumption that NDIS participants will act as informed, rational, and autonomous ‘consumers’ [36]. This can entrench inequities, because not all people have resources to advocate for themselves, the ability to obtain supporting evidence, or access to suitable support providers in areas where markets are ‘thin’ [22].

### Women are underserviced by the NDIS

Yates et al. [43] proposed three reasons why women are underserviced by the NDIS: (1) women are *under diagnosed* with conditions commonly accepted by the NDIA (e.g. autism) while being far more likely to be diagnosed with chronic health or autoimmune conditions that are *less likely to be accepted* by the NDIA; (2) women are socialised to deemphasise their own needs, which may mean women are ‘less effective self-advocates than men’ [43, p.2]; and (3) the gendered nature of caring responsibilities alongside the complications women face in having these responsibilities supported, particularly in relation to parenting [43].

The Scheme uses personalisation of social and healthcare services to support goal setting to ‘live a normal life’ and to set budgets to achieve these goals [11]. While driven to streamline government oversight and emphasise the rights of the individual, personalisation models increase barriers to support for many groups [10, 11]. Women with disabilities report inequitable outcomes in relation to administrative burdens and advocating for individual support [11, 43], especially those from Indigenous or multicultural backgrounds, and those identifying as LBGTQIA + (Lesbian, Bisexual, Gay, Transgender, Queer/Questioning, Intersex, Asexual and other marginalised gender and sexual identities).

A 2019 analysis of inequalities in gendered access to the NDIS conducted by the Scheme administrator [26] concluded that gender disparities evident in participation data are largely the result of an imbalance in support across age categories and disability experiences. The report uses a range of standardisation techniques to conclude that the gender composition of participants reflects a higher prevalence of autism, developmental delay, and intellectual disability among young males. A high proportion of NDIS participants (42%) are children aged 0 to 14 years and a significant proportion of those participants are males accessing autism-related supports [32].

Unfortunately, the NDIA’s 2019 analysis does not address gender bias in access assessments [26]. This is problematic, given the growing awareness of gender bias in diagnosis experiences. For example, autism is more frequently diagnosed in men, with the actual gendered prevalence hard to determine given women tend to be underdiagnosed and more likely than males to be diagnosed later in life [16]. Likewise, the Scheme currently requires diagnosis of a ‘primary disability’ that is named on a NDIS eligibility list. Many of the chronic health and autoimmune conditions that predominantly affect women are not included on these lists [43]. Women are more likely than men to have at least one chronic health condition and are significantly more likely to experience co-occurring conditions [2]. This means women’s experiences of disability are less likely to be recognised, compared to those of men.

Fewer women than men can effectively apply to the Scheme at all. A persistent gender bias in the medical system often results in women’s symptoms being taken less seriously than those of men [21], leading to delayed diagnosis [24] or reduced provision of support [44]. The gendered barriers to diagnosis and the range of conditions predominantly affecting women that are either not included on NDIS eligibility lists or are harder to obtain documentary evidence to support [44], means the scale of unmet need for disability support among women is likely to be significantly higher than the number of declined NDIS access requests would suggest. Although this is an imperfect measure, gendered differences in the handling of access requests across age groups provide a starting point for analysis. Significantly more men are applying for NDIS support, resulting in higher rates of overall participation. The December 2024 Quarterly Report shows similar rates of access approval for male and female children aged 0–14 [32]. However, from ages 15 + male access requests are approved at far higher rates than females and applicants gendered ‘other’. This gendered gap widens for each age band through to 64: in the 15–18 age band the difference between male and female access approvals = 3%; ages 19 to 24 = 5%; ages 25 to 34 = 7%; ages 35 to 44 = 8%; ages 45 to 54 = 10%; and by 55 to 64 the difference is 12% [32, Table E.4]. The gendered patterns in access approval rates across age bands likely reflect normative assumptions about when care/support *for women* is seen as justifiable (under 15, over 65), where women within these age bands (15–65) are normatively positioned as carers *for others* (paid/unpaid) [8]. This foundation informs the theoretical (directive) component of the content analysis of *The Review*, outlined in this paper.

### The [2023]* Review*

*The Review* was initiated to address significant challenges in the design, operations, and sustainability of the NDIS [28]. The findings were informed by almost 4,000 submissions made by people with lived experience of disability, disability organisations, support providers, practitioners, and academics [29]. Roughly 10% of the 10,000 Australians who contributed to *The Review* were identified as people with disability and their families and 2,000 personal stories were catalogued. The collation and interpretation of these submissions was guided by commitments under the UNCRPD [39], Australia’s Disability Strategy 2021–31, and the National Agreement on Closing the Gap, and emphasised co-design with participants and stakeholders.

This approach aimed to restore trust and confidence in the NDIS to effectively support people with disabilities and contribute to broader societal benefits. Importantly, *The Review* committed to prioritising considerations of First Nations participants and ‘participants with a range of *lived experiences* including in relation to *gender*, culture, socio-economic status, age, and sexuality to ensure the NDIS is catering to the diversity of participant needs and intersections between them’ [28; emphasis added]. These considerations contributed to the use of ‘intersectionality’ to frame *The Review*. Unfortunately, *The Review* contains no definition or explanation of what is meant by intersectionality. In the next section, we seek to address that gap.

### Intersectionality

Kimberle Crenshaw, a founding scholar of critical race theory, coined the term ‘intersectionality’ [14]. Since Crenshaw’s seminal analysis of an anti-discrimination case brought about by five Black women against General Motors, the concept has been contested across disciplines, contexts, and in practice [6, 19]. Crenshaw’s analysis identified how the legal system failed to account for the way protected attributes may overlap and, in doing so, entrenched ‘intersecting’ barriers to access that had become embedded in the design of the employment system. Black feminist scholars grounded the theoretical orientation of intersectionality in racism and sexism, which led the expansion of the term to include other systems of oppression, including ableism, classism, homophobia, and transphobia [37].

Crenshaw’s approach demonstrated that hierarchies are maintained through intersecting forms of oppression, which simultaneously reinforce and constitute one another at a *systemic level*. In Crenshaw’s work, processes of domination are reproduced by the employment system, which is mutually reinforced by the legal system that functions as the legitimating authority for decision making. Contrastingly, intersectionality has become a contested term in policy, often diluted to excessively focus on attributes of identity at an *individual level* [19, p.84]. Concerning the present paper, we are interested in parallels between the gender bias of the medical system (the legitimating authority for NDIA decisioning [27]) being reproduced by the disability support system (NDIS). As Yuval-Davis [45, p.195] argues, appropriation of the term conflates and separates the location of intersectionality, rather than understanding the relationship between these divisions.

There are multiple ways in which gender and disability oppression appear to be reproduced by the design of the NDIS, including through reinforcing the gendered bias of the medical system, which is further sanctioned by the language and decisioning of the legal system [41]. Robinson [37, p.478] describes such dilution as ‘oppression as an additive phenomenon’, where structural locations are plotted on effectively static ‘axes’ of oppression within a ‘matrix of domination’ [13]. The scope of this application is limited to overlapping attributes of identity at an *individual level* (e.g. a Black woman), or *group level* (Black women). When public policy adopts this diluted application of intersectionality as a proxy for ‘diversity’ or to describe groups as ‘multiply marginalised’, it obfuscates the *systemic* origins of oppression and contributes to further marginalisation.

Public policy applications that prioritise interpretations of intersectionality at an *individual* or *group level* avoid examination of the ‘multiplicative’ effect of interlocking *systems* of oppression, which paradoxically are often the source of, and sustained by, their own institutional/governmental power. Crenshaw notes: ‘People can only demand change in ways that reflect the logic of the institutions they are challenging’ [15, p.1243]. Therefore, it is difficult to create effective demands for change without understanding the model of dominant ideology.

Addressing the rise of the term in public policy [6], and its appropriation for unintended ends, requires clarifying meaning at a systems level. Systemic discrimination overlaps and compounds experiences of marginalisation intersectionally. Therefore, the notion cannot be used as a proxy for attributes of identity at an individual level. The intelligibility of gendered issues in *The Review* is a case study that reflects how women’s experiences of disability are deprioritised and poorly understood in public policy more broadly. This case study and our methods, described in the following section, provide a framework for recognising and responding to both intersectional and gendered issues within and beyond disability policies in Australia and other jurisdictions.

Recommendation 23.5 of *The Review* states: ‘The Australian Government should ensure that all disability reporting mechanisms facilitate the collection, analysis and publication of intersectional indicators’ [29]. A representative for *The Review* panel told *Crikey*: ‘We understand the barriers faced by women with disability and we call out “intersectionality” specifically’ [46]. To this end, we analyse *The Review* considering the following research question: *What does the 2023 NDIS Review recommend to address gender inequities?*

To answer this question, we investigated:

- How gender inequality was acknowledged and analysed throughout *The Review.*
- How an understanding of gender inequity informed the recommendations.

## Methods

A content analysis of *The Review* was conducted to assess conceptualisation of, and references to, gender. Content analysis allows for both systematic classification and subjective interpretation of texts when identifying themes and patterns in meaning [20, p.1278]. Our approach has been guided by Meltzer and Davy's content analysis that assessed the way relationships were referenced in key NDIS documentation [23]. Following Meltzer and Davy [23], we also used elements of summative and directive approaches as described by Hsieh and Shannon in their typology of options for content analysis [20, p.1286]. The summative component of the content analysis involved the identification of three categories of search terms:

(1) Derivatives of ‘intersectionality/intersectional’.

(2) Gender/ed and familial keywords: ‘female’, ‘male’, ‘women/woman/women’s’, ‘man/men/men’s’, ‘girl/s’, ‘boy/s’, ‘sister/s’, ‘brother/s’, ‘husband’, ‘wife’, ‘father/ dad/s’, ‘mother/mum/s’, ‘son’, ‘daughter’, ‘gender/gendered’, ‘brotherboy/s’, ‘sistergirl/s’, ‘trans/transgender’, ‘non-binary’, ‘gender diverse’ and ‘LGBT/GLBT’.

(3) Keywords that reference gendered disability experiences: ‘abuse/abused/abusive’, ‘violent/violence’ and ‘chronic/CHC’.

Sect. "FINDINGS" of this paper analyses how each instance of these terms was framed and contextualised. The directive component of the content analysis drew on theory and evidence about the importance of disability policy adopting a gendered lens, outlined above (see also [10]).

The terms ‘chronic health’ and ‘violence’ were added as examples of searchable gendered phenomena. Findings in relation to ‘violence’ will be addressed in a separate paper with an expanded content analysis that includes key Disability Royal Commission (DRC) documentation. *The Review* refers heavily to the DRC and, at the time of writing, the government response to this remains under consideration [18]. Chronic health is the gendered phenomenon in focus for this paper, given that earlier reviews recognised it as an unresolved priority area.

Consistent with Hsieh and Shannon’s typology, the key terms reflect the ontological nexus of gender and disability where our research is premised [20]. Analysing the use of these key terms in *The Review* can demonstrate the extent to which gender inequality is written in and out of service development in ways that might reinforce barriers in the provision of inclusive and accessible policy, despite apparent ‘intersectional’ recommendations.

Two documents were selected for the content analysis:

1. Final Report [29]
2. Supporting Analysis [30]

Full copies of the documents in *The Review* were systematically searched to identify all instances of these terms. Using NVivo’s text search, ‘stemmed’ phrases and terms were identified. The full paragraph of text that each key word appeared within was coded by (DKP) under the categories captured within ‘quotation marks’ above. These categories were exported to individual word documents, with others (LH/BE) checking each instance had been captured accurately by referring to the original documents and adding page numbers against each segment of text.

## Findings

In this section we outline the findings of the content analysis, detailing how our three search term categories appear within *The Review*. We begin with intersectionality, before outlining the uses of gendered key words, followed by key words of gendered disability experiences.

### Intersectionality

While ‘intersectional/ity’ is used 93 times in *The Review*, it functions primarily to describe axes of people’s identities that signify overlapping forms of disadvantage (i.e. LGBTIQA+SB identities, gender identities, First Nations identities, culturally and linguistically diverse identities). We identified four main ways in which the term ‘intersectionality’ is operationalised in *The Review*.

#### Intersectionality as a data point

The first way in which intersectionality is understood is as a data point to collect, analyse, and report on various indicators, in recognition of the current lack of information and capacity to disaggregate data. Thus, enhanced data collection is prioritised to capture the various axes of participant identities at an individual level, exemplified in this excerpt:

Gender and sexuality are core elements of identity that impact how people with disability experience all aspects of life, including their disability, diagnosis, interaction with government services (including the NDIS), disability services and supports and social and economic participation. While robust intersectional data is poor, anecdotally we know disability prevalence rates are high amongst LGBTIQA+SB communities. The 2014 ABS General Social Survey estimated that 30 per cent of people who identify as gay, lesbian, bisexual or “other” have a disability. [30, p.62].

While this excerpt acknowledges the disadvantaging effects of gender and sexuality, it fails to explain *how* gender disadvantages. Both gender and sexuality ‘impact’ people’s experiences, but there is no mention of gender inequality – and the explanatory ‘impact’ mentioned here concerns the high disability prevalence rates amongst LGBTIQA+SB communities.

##### Intersectionality as group representation

*The Review* also uses intersectionality to indicate forms of discrimination experienced by specific groups of people, in relation to their diverse and marginal identities. Throughout *The Review*, groups are frequently described as ‘intersectional populations’, as having an ‘intersectional identity’, and as people with ‘intersectional needs’. This approach emphasises marginalised sexualities (and other identity groups) but de-emphasises gender, as shown in this excerpt:

LGBTIQA+SB people with disability face unique stressors due to their intersectional identity that require systemic representation. This includes increased experiences of violence, discrimination, expectations of stigma and concealment of their identities. These factors are linked to increased psychological distress which can exacerbate social isolation and impact socio-economic outcomes such as education attainment, employment and health. [30, p.64].

Here, lack of engagement with gender removes understanding of diverse experiences of sexuality from understanding how marginalisation occurs through gendered inequality.

When *The Review* does note “structural” forms of discrimination and disadvantage, this is usually through the prism of specific groups, for example:

First Nations people with disability, *women with disability*, people from culturally and linguistically diverse and LGBTIQA+SB communities experience intersecting layers of individual and structural discrimination impacting all aspects of their lives. This discrimination means some people are less likely to seek help. [29, p.31; emphasis added].

Even when *The Review* does mention ‘women with disability’, there is no attempt to utilise a gendered lens to understand women’s experiences, despite the importance of the gender/disability nexus supported by theory and evidence described in Sect. "INTRODUCTION". Indeed, this lens would be helpful to incorporate in understandings of *how* ‘intersectional barriers’ are experienced in influencing policy, in the following:

Particular groups of people with disability experience additional barriers in influencing policy. These include children and young people, those with experiences of intersectional barriers and discrimination, people with intellectual and/or psychosocial disability and/or autism, and those who are non-verbal. [29, p.241].

This gap means that when the significant matter of gender inequalities is unnoticed, so is the opportunity to consider reforms to the NDIS to address the persistent, longstanding structural and systemic gendered inequalities that continue to characterise experiences of disability.

Interestingly, despite its heavy use of the term intersectionality, there is only one reference to ‘gendered discrimination’ across *The Review*:

The intersection of racist and ablest attitudes can also contribute to the economic exclusion and high levels of socio-economic disadvantage of First Nations people with disability … This is heightened for First Nations women with disability due to the addition of gendered discrimination in broader society. [30, p.122].

However, there is no attempt to unpack the impacts of gendered discrimination, nor the ways that the NDIS and medical systems systematically reproduce gendered inequalities for First Nations women with disability, or for women and people with disabilities broadly.

##### Intersectional practice – reshaping the subjectivities of frontline staff

The recommendations of *The Review* pronounce intersectionality as a key element for practice design and capacity building. In context, this means that services will be redesigned with an understanding of intersectionality as an innovation in ‘professional development’ – a lever to influence the attitudes of frontline NDIA staff. Intersectionality then becomes a critical element of ‘best practice’, exemplified in *The Panel’s vision: A highly skilled workforce across all areas of disability policy, regulation, service delivery and leadership*:

Professional development for all staff should cover disability awareness, intersectionality and trauma informed practice. It should also include reflective practice (examining what worked well and ensuring it is built into future practice) to ensure all staff have the skills and experience to meet the needs of the people they serve. [29, p.258].

However, this is arguably an additive approach, rather than transformative. It appears that competencies in intersectional analysis will be ‘added’ to the skills of frontline practitioners, akin to an upgraded version of ‘diversity and inclusion’ training. The benefit, according to *The Review*, is to demonstrate awareness and analysis of individual service users, rather than utilising understandings of inequality, inclusive of gender, in case-based interactions: ‘these assessors must have the knowledge and empathy to recognise and respond appropriately to intersectional needs and multiple disabling conditions’ [30, p.290].

Within this individualised approach, frontline workers are called upon to ‘meaningfully consider how core characteristics such as gender, sexuality, age, cultural and religious beliefs intersect to impact needs’. Here, the term ‘intersectional’ is used to describe overlapping needs and disability experiences [30, p.275]. The recommendation for this ‘lens’ to be ‘tested with intersectional community leaders’ is striking because, again, it individualises the intersectionality priority in *The Review* by transferring responsibility to these ‘leaders’ to ensure the effectiveness of the new initiatives, reflective of neoliberal processes [33].

##### Intersectional structural reform

When intersectionality is used in terms of ‘structural reform’, the interventions in the system may again be viewed as ‘additive’ rather than systemic. The lack of attention to break down identities captured within these proposals, and the conceptualisation of different axes of identity experienced through individual-level needs, neglects the intersecting nature of systemic biases, resulting in inadequate support for marginalised groups. For example, Action 2.1 proposes the development of a Foundational Supports Strategy, which will include a ‘dedicated advisory group made up of Disability Representative Organisations and people with disability … including representation from intersectional groups, including First Nations people, culturally and linguistically diverse, women and LGBTIQA+SB’ [30, p.40].

*The Review* also recommends that disability legislation be ‘reviewed and improved’ to establish a more consistent and effective approach across all Australian jurisdictions to ‘tackle discrimination’, including ‘a positive duty to promote inclusion, establishing a responsible Inclusion Commissioner to monitor progress, universal design and considerations of intersectionality and ableism’ [30, p.123]. Although *The Review* mentions discrimination, there is no evidence within the documents to suggest that an Inclusion Commissioner would be aware of the significant gender inequities apparent for women with disabilities within Australia. The overriding focus of these reforms is on broadening the inclusiveness of the NDIS at a sectoral level, which is a positive move. However, it does not question—or address—the way in which the design and operation of the NDIS perpetuates gendered disadvantage.

### ‘Women’ and gendered familial terms

Although appearing 41 times throughout *The Review*, the term ‘women/woman’ is used narrowly. To extend this analysis, this section compares the context where gendered familial terms appear, examining differences in uses of derivatives of ‘father/husband/son’ compared with their female equivalents ‘mother/wife/daughter’. Derivatives of ‘women/woman’ appear in two clear patterns. Firstly, when quoting submissions to *The Review* from ‘Women with Disabilities Australia’ (WWDA). However, all six quotations from WWDA relate to plan administration and market considerations that could be interpreted for a general audience, rather than citing any specifically gendered content. Secondly, ‘women’ appears in general statements made about marginalised groups, for example: ‘First Nations people with disability, *women*, culturally and linguistically diverse and LGBTIQA+SB communities’ [30, p.122, emphasis added]. Following our analysis of the key term ‘intersectionality’, this contributes to the additive approach of understanding representation and inclusion in *The Review*, rather than contributing to understandings of structural inequalities. Indeed, this becomes clear as the final recommendations include explicit considerations for each of these groups, except women with disabilities specifically.

The final recommendations justifiably prioritise better supporting First Nations people (recommendations 2.10, 14.1, 20.4), understanding culturally diverse concepts of disability and care (recommendation 2.2) and systemic advocacy for LGBTQIA + SB people (recommendation 1.6). Along with these groups, *The Review* acknowledges women with disability face barriers to receiving help [29, p.31] and efforts to understand their experiences ‘need to be accelerated’ [30, p.1116]. Unlike these groups, though, there are no specific recommendations that relate to women.

In contrast, ‘women’ are visible in *The Review* through their relationships and caring responsibilities, demonstrated by gendered familial terms. *The Review* makes several welcome recommendations that acknowledge ‘families and caregivers’ (recommendations 1.8, 4.4, 6), but again, none of these are explicitly gendered. Within the detail of the supporting analysis, *The Review* briefly acknowledges the gendered nature of *paid* care, noting 7 in 10 of the 280,000 disability support workers in 2021–22 were female [30, p.849]. Glaringly, there is no breakdown or explicit commentary concerning the gendered nature of *unpaid* (often familial) care, when in Australia 72% of primary carers are female [5].

In stark contrast, there are clear patterns in gendered familial terms used in illustrative case studies and excerpts from submissions. Notably, whenever male familial terms ‘father/dad/husband/brother/son’ were positioned in carer roles, these caring responsibilities were shared with a ‘mother/mum/wife/sister/daughter’ (female familial terms) counterpart. For example: ‘I rely on others to advocate for me, mainly mum and dad, as it is almost impossible to get an advocate’ [30, p.60]. In this statement, the participant quoted relies on *both* parents for support. This pattern is reproduced in the (fictional) illustrative case studies included throughout *The Review*, e.g.: ‘Henry, 35 years old, diagnosis of Intellectual Disability, Autism Spectrum Disorder and Epilepsy. Henry lives with his mother and father in a very large regional centre. His parents are now in their late 60s’ [30, p.300]. It is worth noting, while ‘mother and father’ are named together here, the broader case study positions ‘Henry’s mother’ as the liaison between her son and the NDIA. This indicates the fictional case studies provided are potentially overstating the shared caring roles, further obscuring their gendered prevalence. There is only one instance where a ‘caregiving’ brother is referred to in isolation and it is to illustrate a case of fraud where said brother is being removed as plan nominee [30, p.383]. Conversely, there are at least five separate instances where female familial terms are positioned in caring roles, without the mention of other familial support. There are attributions to ‘carers’ and ‘parents,’ and these may potentially include men who are in solo caring roles.

### Gender inequity in NDIS servicing

This section addresses gender disparities within NDIS participation by examining terms related to trans/gender experiences and chronic health conditions. We examine these terms in the context of Yates and colleagues’ [43] analysis of why women are underserviced by the NDIS (see Sect. "Women are underserviced by the NDIS"). There is a dearth of research available on barriers to NDIS access for transgender people. This area warrants *standalone* analysis, however, for this study, we examined the visibility of both transgender and female gender labels *together*.

Following the narrow references to ‘women’s’ experiences, derivatives of ‘female’ are similarly sparse, with only five appearances throughout *The Review*. The words ‘transgender’ or ‘non-binary’ appear nowhere in the documents, however ‘trans-identifying’ appears once, with a focus on autism: ‘In Australia, a recent study found that 22.5 per cent of trans-identifying people have an autism diagnosis, compared to 2.5 per cent of the Australian population’ [30, p.62]. There are 12 mentions of ‘gender diverse’/ ‘gender and sexually diverse’ people/communities, two are again in relation to experiences of autism. In the remaining 10 instances [30, p.66; p.969], *The Review* does not apply terms with any depth, rather it appears as an ‘add on’ to other descriptors, evidencing the ‘additive’ approach to intersectionality discussed previously.

In contrast, the key terms LGBT and GLBT are referenced 56 times across *The Review*. This is most evident in recommendation 1.6, which states:* ‘*All Australian governments should fund systemic advocacy of LGBTIQA+SB people with disability to strengthen representation at all levels’. It is hard to determine if the ‘ + SB’ denotation to include ‘brotherboy and sistergirl’ in the acronym is understood by *The Review*. Within both Glossaries of *The Review*, LGBTIQA+SB is defined as: ‘Lesbian, Gay, Bisexual, Intersex, Queer or Questioning, Sistergirl and Brotherboy’, thereby excluding the ‘T’ for transgender. Definitions provided for the terms ‘Brotherboy and Sistergirl’ deemphasise their necessary relationship to *gender*, by describing them as ‘culturally distinct *queer* identities in First Nations communities’. Taken together with the sparse attention to gender experiences throughout *The Review* generally, this indicates lack of clarity on how members of the disability community meaningfully understand and experience gender.

While a dedicated section of *The Review* addresses ‘The inequity of access to the NDIS’, only a small portion addresses the gendered nature of inequities [30, pp.224–5]. It does not extend any further than the NDIA’s 2019 report on gender and the NDIS, except to acknowledge that people gendered ‘other’ have the lowest access approval rates [26]. The already unmet need for specialised advocacy is heightened with *The Review’s* proposal to remove Access Lists.

The ‘gate-opener’ to be considered for NDIS support in the current system requires a person’s disability to be named on an Access List. Many chronic health conditions that are disproportionately diagnosed in women are *not* included on these lists, which automatically denies them the opportunity to have their support needs considered. Removing Access Lists may have the potential to expand avenues for women to access support. However, in the current system even *listed* chronic health conditions are increasingly declined, with *The Review* confirming: ‘access met rates for adult applicants with CHC [chronic health conditions] have declined strongly since mid-2020 and are reaching very low levels (25 per cent per quarter as per end of 2022) relative to non-CHC applicants (73 per cent)’ [30, p.74]. *The Review* does not include a gendered breakdown to illustrate the impact of this decline in chronic health-related access requests.

Despite this known gendered prevalence of CHC, *The Review* confirms only: ‘Adults with chronic health conditions make up over half of all Australians who have not met access for the NDIS. Since the Scheme’s inception, as of September 2022, around 56,000 people with chronic health conditions as a primary condition have applied and been deemed ineligible’ [30, p.29]. It is reasonable to infer that access denials related to CHCs have disproportionately affected women and contributed to their overall underrepresentation within the Scheme. While *The Review* recommends removing Access Lists in favour of functional assessments, it does so without addressing how the proposed new system will avoid exacerbating existing gendered barriers.

## Discussion

The NDIS Review assembled a ‘Co-Group’ comprised of members from key Disability Representative Organisations. A requirement to develop *a gender strategy* featured in the Co-Group’s ‘recommendations for intersectionality’ [30, p.1199]. This strategy is not mentioned and none of its content is evident in *The Review* recommendations. At minimum, the Co-Group’s requirements would suggest that gender should be integral to the analysis incorporated into the final review. However, as is clear from the findings presented above, attention to gender is notably lacking. This might be the result of a decision made in *The Review* to incorporate gender under the term intersectionality. This should allow for a more nuanced account of the multiple intersecting forms of oppression, which simultaneously reinforce and constitute one another at a *systemic level* to shape unequal access to NDIS supports and services.

Unfortunately, the NDIS Review shows minimal engagement with either gender or intersectionality. Rather, *The Review* includes a diluted application of intersectionality where it is applied variably as an ‘marker’ of marginalised individual and group identities, a ‘product’ for training individual service staff, or a marketing term to ‘sell’ policy change. This narrows the analytical lens applied in *The Review*, which conceals the institutional reforms necessary to address gender inequities.

The NDIS Review’s understanding of gender has largely been displaced with frequent misuse of the term ‘intersectionality’ throughout its documents. Despite a thick Glossary in both key documents of *The Review*, neither offers a definition of intersectionality. As discussed above, intersectionality must involve examination of the way gender and racial bias are embedded in the design of systems, and mutually reinforced and legitimatised by other systems. This may be observed, for example, in the way NDIS rules applied in practice result in significant participation disparities between cisgendered men/young boys and others [32]. Decisions are anchored to forms of medical evidence [27] and processes that effectively limit access for women [43] and culturally and racially marginalised people [12], thus reproducing the gendered bias of the medical system [7].

The removal of Access Lists is insufficient without attention to systemic gender bias. Importantly, there are chronic health conditions that disproportionately impact women, which are explicitly named on Access Lists in the existing system including: Multiple Sclerosis, where 75% of people diagnosed are women [25]; Lymphedema, where 69% of cases severe enough to result in hospitalisations are women [3]; and Rheumatoid Arthritis, which affects 2.5% of females and 1.6% of males [4]. Even with these diagnostic gate openers met, *The Review* confirms NDIS access approvals for CHCs remain 48% lower than access requests made for non-CHC related disability [30, p.74].

To be genuinely intersectional, the measures to quantify functional impairment and determine access to NDIS supports must account for gender. Otherwise, the gendered bias of the medical system will continue to be reproduced and legitimated by the design and operations of the NDIS. This conclusion is not inevitable. It reflects, firstly, which presentations of disability are determined worthy of support and, secondly, political priorities. For example, many CHCs experienced predominantly by women are characterised by fluctuating symptoms. Disability that is episodic, meaning it does not always present in the same way is not well understood by the NDIS, and exacerbates the already relentless demands for self-advocacy from participants [43]. *The Review* reflects political priorities for market regulation, rather than systemic transformation. Taken together, the lack of attention to addressing gender bias and contingent policy co-ordination results in ‘care systems’ that entrench inequality. The hurdles women navigate to access support paradoxically result in a displacement of intended care.

In its rhetoric, both documents demonstrate *The Review’s* responsiveness to the specific needs of ‘intersectional groups’, but the proposed responses do not account for, or address, broader systemic patterns of gender inequality. Addressing ‘intersectionality’ requires more than collecting data about the individual identities of participants. In this sense, ‘intersectionality’ becomes a marketing buzzword to signal a socially just approach and ‘sell’ policy change to marginalised groups. In this new operating model, intersectional sensitivity is to be demonstrated at every level of service provision, through taking a ‘deliberately intersectional approach’ [29, p.32] and developing ‘tailored’ models to represent the specific needs of intersectional cohorts, seen as part of the rebuilding of the ‘disability ecosystem’ [29, pp.204, 227, 242]. However, this approach is ‘additive’ [37], rather than transformative and, as we assert, can continue to create harm for people with disabilities. It seems that considerations of intersectionality are largely conceived as an element to check off for compliance, while the fundamental structure of the system remains unchanged.

Avoiding the gendered dimension of disability resource allocation disregards the complexities of parenting, gendered medical bias, and the disadvantages women experience in schemes predicated on self-advocacy. Furthermore, these phenomena manifest in culturally specific ways that warrant direct attention. *The Review’s* application of intersectionality, instead, positions ‘additive’ axes of marginalisation as the gate-opener for discussion, while completely ignoring the scale of gendered issues that are experienced commonly across cultural groups.

*The Review’s* interpretation of intersectionality acknowledges different axes of identity of the *individual*, but not *intersecting systems* of oppression. Even when ‘systemic’ references are made to intersectionality, this is not about systemic reform in practice. It amounts to little more than provisions for committees (who are removed from any real decision-making power) and adding ‘people who are different’ to the existing system. Ultimately, any structural reforms discussed focus on alterations via market mechanisms (e.g. provider regulation), and position intersectionality as a ‘brand’ into which policy ‘products’ can be packaged.

## Limitations and future directions

This paper has undertaken an analysis of how *The Review* has understood gender. The findings comprehensively demonstrate the absence of attention to *women’s experiences*. To avoid the ‘additive’ approach to intersectionality that this paper critiques, we have chosen to be clear about the scope of analysis to report findings that are in-depth and specific. There are limitations to interpreting our analysis at the aggregate level of *all marginalised gender experiences*. There are similarities in the inequities experienced broadly by ‘people who do not identify as cisgender men’ that apply to our present analysis. However, barriers to access manifest differently between people who are non-binary/trans-identifying and people who are female. Further research must focus specifically on the differences *within* the broad constellation of transgender experiences. Likewise, *the Review’s* diluted application of intersectionality and lack of systemic focus limits its transformational potential in addressing racialised discrimination and inequity within the NDIS. The experiences of First Nations people and migrant and refugee people with disabilities must therefore remain a specific focus in research agendas addressing barriers to NDIS access.

## Conclusion

*The Review’s* Terms of Reference [28] expressly aimed to prioritise lived experience, but it is questionable to what degree this has translated into the final recommendations. Our analysis of *The Review* reveals that gender inequality has all but disappeared from consideration, in favour of a diluted application of ‘intersectionality’. As a concrete site for analysis, *The Review* reflects broader calls for clarity and consistency when operationalising key terms in public policy [6]. ‘Intersectionality’ and its derivatives appear within *The Review* as a proxy for concepts that could be more aptly labelled as ‘diversity’ and ‘overlapping’/compounded’, or ‘co-occurring’ (e.g. axes of identity, disability experiences and interactions between service delivery departments/arms of government, etc.). In these diluted applications, ‘intersectionality’ is removed from its origins in critical theory. These muddied applications are not only insufficient to address gender; ultimately, they obscure the systemic failings experienced by all marginalised groups.

## Data Availability

The NDIS review documents analysed within the manuscript are publicly available from https://www.ndisreview.gov.au/resources/reports/working-together-deliver-ndis.

## Footnotes

1 This paper uses ‘person first’ language (women/people with disabilities) in alignment with People with Disabilities Australia’s language guide [35]. We acknowledge people describe their experience of disability in different ways and, for many people, ‘identity first’ language is a source of pride and resistance.
